# 408 Cases of Genital Ambiguity Followed by Single Multidisciplinary Team during 23 Years: Etiologic Diagnosis and Sex of Rearing

**DOI:** 10.1155/2016/4963574

**Published:** 2016-11-28

**Authors:** Georgette Beatriz De Paula, Beatriz Amstalden Barros, Stela Carpini, Bruna Jordan Tincani, Tais Nitsch Mazzola, Mara Sanches Guaragna, Cristiane Santos da Cruz Piveta, Laurione Candido de Oliveira, Juliana Gabriel Ribeiro Andrade, Guilherme Guaragna-Filho, Pedro Perez Barbieri, Nathalia Montibeler Ferreira, Marcio Lopes Miranda, Ezequiel Moreira Gonçalves, Andre Moreno Morcillo, Nilma Lucia Viguetti-Campos, Sofia Helena Valente Lemos-Marini, Roberto Benedito de Paiva Silva, Antonia Paula Marques-de-Faria, Maricilda Palandi De Mello, Andrea Trevas Maciel-Guerra, Gil Guerra-Junior

**Affiliations:** ^1^Interdisciplinary Group of Study of Sex Determination and Differentiation (GIEDDS), School of Medicine (FCM), State University of Campinas (UNICAMP), Campinas, SP, Brazil; ^2^Department of Pediatrics, FCM, UNICAMP, Campinas, SP, Brazil; ^3^Growth and Development Laboratory, Center for Investigation in Pediatrics (CIPED), FCM, UNICAMP, Campinas, SP, Brazil; ^4^Laboratory of Human Molecular Genetics, Center of Molecular Biology and Genetic Engineering (CBMEG), UNICAMP, Campinas, SP, Brazil; ^5^Laboratory of Clinical Pathology, Clinical Hospital, FCM, UNICAMP, Campinas, SP, Brazil; ^6^Pediatric Surgery, Department of Surgery, FCM, UNICAMP, Campinas, SP, Brazil; ^7^Cytogenetics Laboratory, Department of Medical Genetics, FCM, UNICAMP, Campinas, SP, Brazil; ^8^Department of Medical Genetics, FCM, UNICAMP, Campinas, SP, Brazil

## Abstract

*Objective*. To evaluate diagnosis, age of referral, karyotype, and sex of rearing of cases with disorders of sex development (DSD) with ambiguous genitalia.* Methods*. Retrospective study during 23 years at outpatient clinic of a referral center.* Results*. There were 408 cases; 250 (61.3%) were 46,XY and 124 (30.4%) 46,XX and 34 (8.3%) had sex chromosomes abnormalities. 189 (46.3%) had 46,XY testicular DSD, 105 (25.7%) 46,XX ovarian DSD, 95 (23.3%) disorders of gonadal development (DGD), and 19 (4.7%) complex malformations. The main etiology of 46,XX ovarian DSD was salt-wasting 21-hydroxylase deficiency. In 46,XX and 46,XY groups, other malformations were observed. In the DGD group, 46,XY partial gonadal dysgenesis, mixed gonadal dysgenesis, and ovotesticular DSD were more frequent. Low birth weight was observed in 42 cases of idiopathic 46,XY testicular DSD. The average age at diagnosis was 31.7 months. The final sex of rearing was male in 238 cases and female in 170. Only 6.6% (27 cases) needed sex reassignment.* Conclusions*. In this large DSD sample with ambiguous genitalia, the 46,XY karyotype was the most frequent; in turn, congenital adrenal hyperplasia was the most frequent etiology. Malformations associated with DSD were common in all groups and low birth weight was associated with idiopathic 46,XY testicular DSD.

## 1. Introduction

One of many possible medical emergencies in newborns is genital ambiguity that has significant importance both immediately after birth, such as congenital adrenal hyperplasia and certain malformation syndromes that may present potential risk to child's life, and in the long term, such as unresolved sex definition that may cause irreversible psychosocial effects for patients and their families [1, 2]. An experienced multidisciplinary team is required for the proper care of children with ambiguous genitalia, which is usually found in tertiary and university care centers [1–3].

The incidence of disorders of sex development (DSD) is not fully known. In 2000, Fausto-Sterling suggested that it corresponds to 1.7% of live births [4]. However, two years later Sax questioned this estimative arguing that the author had included patients without genital ambiguity, such as those with Turner and Klinefelter syndromes and the nonclassical form of congenital adrenal hyperplasia and suggested that the incidence of DSD with genital ambiguity would be actually 0.0018% [5].

The Chicago Consensus in 2006 [1], updated in 2016 [2], established a classification for DSD based on karyotype. However, some authors have questioned this classification and proposed a different classification based on type of gonadal tissue [6–9].

Therefore it is evident that several issues on DSD are still under discussion. In order to add insights to the DSD study, such as classification, frequency of different diagnoses, sex definition, and sex reassignment, the aim of this study was to describe the experience of the Interdisciplinary Group of Study of Sex Determination and Differentiation (GIEDDS) in the School of Medicine (FCM) and Clinical Hospital (HC) at the State University of Campinas (UNICAMP), Brazil, over 23 years in the care of newborns, children, adolescents, and adults with DSD and genital ambiguity, analyzing the frequency of each cause, the age at diagnosis, the sex of rearing, and reassignment.

## 2. Materials and Methods

All cases of ambiguous genitalia seen at the Outpatient Clinic of GIEDDS, FCM, HC, UNICAMP, between January 1989 and December 2011 were included in the study. During this period, the same medical team of pediatric endocrinologists, geneticists, psychologists, and pediatric surgeons had followed every case. Karyotyping had been performed in the Cytogenetics Laboratory in the Department of Medical Genetics at FCM, UNICAMP, scoring at least 30 metaphases. Hormonal and biochemical tests were performed in the Laboratory of Physiology at HC, UNICAMP. Molecular tests were performed at the Laboratory of Human Molecular Genetics at the Center of Molecular Biology and Genetic Engineering (CBMEG), UNICAMP.

The criteria used for the definition of genital ambiguity were those set by the Chicago Consensus in 2006 [1]. The cases were classified in four major groups based on karyotype and gonadal tissue: (1) disorders of gonadal development (DGD) irrespective of the karyotype; (2) 46,XX ovarian DSD; (3) 46,XY testicular DSD; and (4) complex malformations of the external genitalia, defined in this study as others (see Supplementary Material available online at http://dx.doi.org/10.1155/2016/4963574).

We evaluated the following data: age (in months) at the first visit, birth weight (in grams), social sex at the first and last visit, and etiologic diagnosis.

Data were stored in SPSS 16.0 spreadsheets and presented as absolute and relative frequency. The Mann-Whitney test was used to analyze age differences between the four major groups of diseases, with *α* = 0.05.

## 3. Results

Over the 23 years of the study, our team attended 408 cases of genital ambiguity. Among them, 189 (46.3%) had 46,XY testicular DSD, 105 (25.7%) 46,XX ovarian DSD, 95 (23.3%) a DGD, and 19 (4.7%) other complex malformations (Table 1). Regarding the karyotypes, 250 (61.3%) were 46,XY, 124 (30.4%) were 46,XX, and 34 (8.3%) had numerical or structural abnormalities of sex chromosomes with or without mosaicism.

Among the 105 cases of 46,XX ovarian DSD, the great majority had congenital adrenal hyperplasia (*n* = 69, 65.7%); 68 had 21-hydroxylase deficiency distributed between simple virilizing form including one case with associated 45,X/46,XX Turner syndrome (*n* = 17) and salt-wasting form (*n* = 51) and one case of P450 oxidoreductase deficiency; all had molecular confirmation. Among patients with syndromic features (*n* = 10, 9.5%), three had VATER association, one had Seckel syndrome, one had Wolf-Hirschhorn syndrome [46,XX,del(4p)], one had caudal regression, and four remained with unknown etiology (Table 1).

In the 46,XY testicular DSD group (*n* = 189), most were syndromic (*n* = 40) or idiopathic (*n* = 77). It is relevant to mention that 42 idiopathic cases (22%) presented birth weight < 2,500 g (from 700 to 2,500 g; mean = 1,934 g). Regarding the group with a defined diagnosis, androgen receptor defects (*n* = 25), 5*α*-reductase type 2 deficiency (*n* = 20), and hypogonadotropic hypogonadism (*n* = 12), including six with associated hypopituitarism, were found with higher frequencies, followed by the group of defects in testosterone synthesis that included one patient with HSD3B2 deficiency, one with CYP17A1 deficiency, and two with HSD17B3 deficiency. All of them had their diagnosis confirmed by molecular studies. Cases of syndromic features included VATER association (*n* = 3), CHARGE syndrome (*n* = 3), Aarskog syndrome (*n* = 3), foetal alcohol syndrome (*n* = 3), Robinow syndrome (*n* = 1), GBBB syndrome (*n* = 1), Noonan syndrome (*n* = 1), and four with autosomal chromosome abnormalities [46,X,add(1)(q43), 46,XY,del(4p), 46,XY,add(10)(q26), 46,XY,t(13;14)(q11;q11)]; 21 remained with unknown etiology (Table 1).

Regarding 95 cases included in the DGD group, there was a predominance of partial and mixed gonadal dysgenesis, followed by ovotesticular DSD. The distribution of different karyotypes in ovotesticular DSD was 46,XX (*n* = 8), 46,XY (*n* = 6) and 46,XX/46,XY (*n* = 2); other karyotypes such as 47,XXY, 46XX/47,XXY/48,XXYY, 45,X/46,XY, 45,X/47,XYY, 45,X/46,X+mar(Y+), and 45,X/47,XY,+mar(Y+) were also found (*n* = 1, resp.). Different karyotypes were also observed within the group of patients with mixed gonadal dysgenesis. From a total of 25 patients 12 were 45,X/46,XY, and the remainders had numerical and/or structural abnormalities: 45,X/46,X+mar(Y+) (*n* = 4), 45,X/46,XY/47,XY,+mar(Y+) (*n* = 3), and 45,X/46,Xi(Yq), 45,X/46,X,del(Yq), 45,X/46,X,idic(Yq), 45,X/46,Xi(Yq)/47,Xi(Yq),i(Yq), 45,X/46,Xi(Yq)/46,X,r(Y)/47,Xi(Yq),r(Y), and 45,Xinv(9)(p13;q21)/46,XY,inv(9)(p13) (one case of each). Among patients with testicular regression syndrome, one had bilateral agonadism and six had bilateral anorchia (Table 1).

Finally, the distribution of the diagnosis for the remaining 19 cases was as follows: five cases of penis malformation including partial penoscrotal inversion (*n* = 2), penis agenesis (*n* = 1), abnormal penis rotation (*n* = 1), and penoscrotal adherence (*n* = 1); clitoral malformation included clitoris agenesis (*n* = 4), severe clitoris hypoplasia (*n* = 1), and lipoma on the clitoris (*n* = 1); diagnosis in cases with multiple complex malformations included exstrophy of the cloaca and cardiomyopathy (*n* = 2), Prune-Belly syndrome (*n* = 1), and perineal lipoma (*n* = 1) (Table 1).

**Figure 1:**
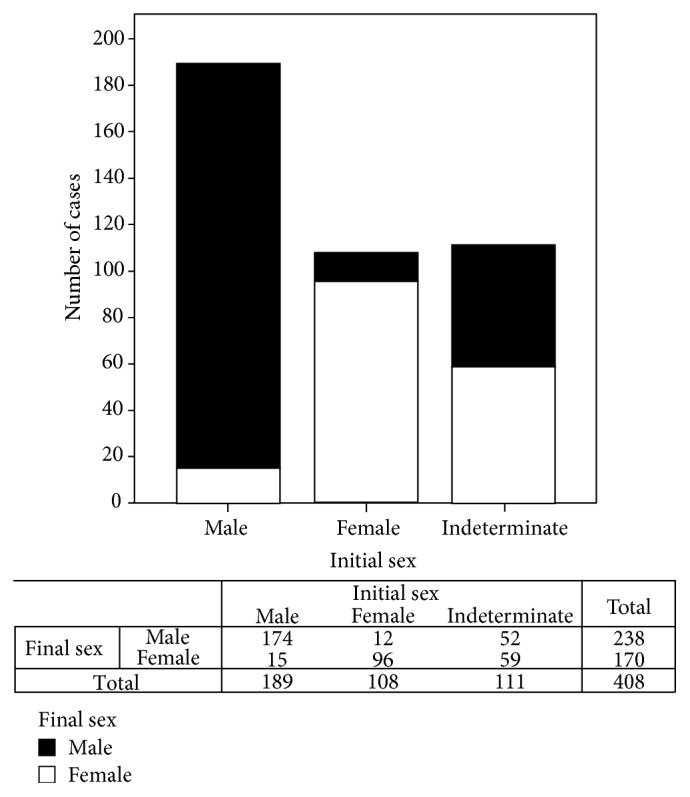
Distribution of initial and final sex assignments in 408 consecutive cases of DSD with ambiguous genitalia followed at GIEDDS, UNICAMP, between January 1989 and December 2011.

Analysis of sex definition in the 408 patients with ambiguous genitalia revealed that 111 (27.2%) patients came to the first consultation without sex definition; after clinical and laboratorial investigation 52 were assigned as male and 59 female (Figure 1). We also observed that 15 cases from the total of 189 who had initial registration as male were reassigned as female; from 108 who had initial registration as female 12 were reassigned as male (Figure 1). Therefore, 6.6% (27 cases) had sex reassignment, all of the them before one year of age, except for the patients with 5*α*-reductase type 2 deficiency.

Table 2 shows the distribution according to initial and final sex and clinical and laboratorial diagnosis. Considering those 15 cases that were initially registered as male and were reassigned as female, seven had 46,XX ovarian DSD due to congenital adrenal hyperplasia, four had mixed gonadal dysgenesis, three had ovotesticular DSD, and one had 46,XY idiopathic testicular DSD. In turn, among the 12 females who were reassigned as males seven had 5*α*-reductase type 2 deficiency, two had partial gonadal dysgenesis, one had mixed gonadal dysgenesis, one had ovotesticular DSD, and one had syndromic 46,XY testicular DSD. All cases of 46,XX ovarian DSD had female final sex assignment. In the 46,XY testicular DSD group, the great majority had male final sex assignment, except for cases of LHCG receptor defect, defects of testosterone synthesis, and complete androgen insensitivity. Within the DGD group, there was not a predominance of either sex, except for 46,XX testicular DSD and testicular regression syndrome (except one case of agonadism), all of them assigned as males. For other causes of genital ambiguity, in general, the final sex followed the genotypic sex.

One hundred and ninety-three (47.3%) out of 408 cases had the first visit in GIEDDS, UNICAMP, before six months of age (Table 2). The distribution according to karyotype was 46,XX (*n* = 82), 46,XY (*n* = 95), and numerical or structural abnormalities of sex chromosomes (*n* = 16). Among them, 105 did not have a sex assignment; 51 were male and 37 female; 95 and 98 had final sex assignment as male and female, respectively. In this group the diagnosis was as follows: 46,XX ovarian DSD (*n* = 74), 46,XY testicular DSD (*n* = 74), DGD (*n* = 37), and other malformations (*n* = 8).

The average age at the first visit was 31.7 months (about three years); it was significantly lower in the 46,XX ovarian DSD group than in the group of 46,XY testicular DSD (Mann-Whitney test, *p* < 0.0001) and DGD (Mann-Whitney test; *p* < 0.0001), but it did not differ significantly from the group of other malformations (Mann-Whitney test, *p* = 0.06). The 46,XY testicular DSD group did not differ from the DGD group (Mann-Whitney test, *p* = 0.975) and from the group of other malformations (Mann-Whitney test, *p* = 0.125); the DGD group did not differ from the group of other malformations (Mann-Whitney test, *p* = 0.19) (Table 3).

## 4. Discussion

This is a descriptive study with a large case series of DSD with genital ambiguity, investigated over a long period by the same clinical and laboratory staff. To our knowledge, this is the largest consecutive series of DSD cases with genital ambiguity under a single service in the literature.

Over 23 years, 408 cases of genital ambiguity had been studied with a predominance of 46,XY karyotype (61.3%), twice as frequent as 46,XX (30.4%). Numerical or structural abnormalities of sex chromosomes with or without mosaicism were found in less than 10%. Our results were similar to those described by Cox et al. [10], who analyzed 649 cases of an international register and found that 460 (71%) were 46,XY and 121 (19%) 46,XX and 68 (10%) had sex chromosomes abnormalities, confirming that DSD with genital ambiguity are more frequent in patients with 46,XY karyotype, due to the complexity of male sexual differentiation [11].

One-fourth of the total number of patients had a diagnosis of 46,XX ovarian DSD, mainly congenital adrenal hyperplasia due to 21*α*-hydroxylase deficiency, confirming the high frequency of this disease in all DSD samples [10, 12]. The frequency of the salt-wasting form was also similar to that found in several population studies [13], confirming that it is the most relevant etiology of DSD due to both its high frequency and the high risk of death. Isolated clitoromegaly and syndromic features were also observed, which shows the importance of a wide range of clinical, hormonal, and molecular investigation for DSD, as well as the significance of the finding of dysmorphisms and associated malformations in cases of genital ambiguity [10, 14, 15].

When it concerns DSD with 46,XY karyotype, there are many challenges for the medical staff and for patients and families, because male sexual differentiation is complex and many differential diagnoses require time and experienced medical staff to establish an accurate diagnosis as quickly as possible [11]. Almost half of the cases (189/408) were of 46,XY testicular DSD and within this group prevalent diagnoses were those of defects in androgen receptor (both partial and complete forms) and 5*α*-reductase type 2 deficiency [16–18]. Such differential diagnoses are challenging in the newborn, especially when there is no history of consanguinity or similar cases in the family [16, 19, 20], which challenges sex definition for those children [21]. Furthermore, among 46,XY testicular DSD, we should also highlight cases of hypogonadotropic hypogonadism with or without hypopituitarism, in which the presence of micropenis with cryptorchidism and without hypospadias strongly suggests this diagnosis [22]. Among less frequent etiologies of 46,XY testicular DSD with a monogenic origin there were defects in the LHCG receptor and in testosterone synthesis and persistence of Müllerian ducts. Even less frequent were those cases associated with the use of drugs by the mother during pregnancy. On the other hand, among the most frequent cases in the 46,XY DSD testicular group were those with syndromic features, once again showing the importance of assessment of dysmorphic features of these patients [10] and the idiopathic forms. In the latter, half of the patients weighed less than 2,500 grams at birth. The association of small for gestational age and genital ambiguity in newborns with 46,XY karyotype is frequently reported in the literature [10, 15, 23, 24], but the explanation is still unclear.

Fetal gonadal determination is influenced by many genes (*SRY*,* NR5A1*,* WT1*,* SOX9*, etc.) and may be disrupted by abnormalities in sex chromosomes [8–11]. With techniques of genomic sequencing, new genes are emerging as participants in the complex process of gonadal development [25]. The DGD group includes diseases in which gonadal differentiation was inadequate or incomplete; therefore diagnoses required histological confirmation by an experienced pathologist [26]. For diseases associated with chromosomal and genetic abnormalities, it was necessary to perform laboratorial tests using conventional cytogenetic, molecular cytogenetic, and molecular genetic techniques [27]. Among 95 cases of DGD, we observed a prevalence of the 46,XY karyotype, followed by sex chromosomes abnormalities; in turn, the 46,XX karyotype was less frequent. Among DGD cases, partial gonadal dysgenesis was the most frequent, followed by mixed gonadal dysgenesis and ovotesticular DSD. Mutations were identified in a specific gene in only 16 out of the 39 cases of partial gonadal dysgenesis [28–33]. The identification of a mutation in the specific gene is important for the follow-up of these cases, particularly mutations in* WT1* that are associated with risk of kidney cancer and gonadal and renal failure [30, 31].* NR5A1* mutations are associated with risk of adrenal insufficiency and primary ovarian failure [32–34]. In a family with mutation in* SRY*, there was a wide spectrum of XY gonadal dysgenesis manifestation, varying from partial to complete forms [28]. Other cases with no known mutations are candidates for genomic approaches [25]. In cases of partial gonadal dysgenesis the prognosis of spontaneous puberty in patients assigned as males is relatively good [35]. Cases of mixed gonadal dysgenesis, mainly those with 45,X mosaicism, require follow-up for diseases associated with Turner syndrome and short stature [36]. Cases of ovotesticular DSD, which may occur in the presence of any chromosomal constitution, represent a challenge for the sex of rearing definition, prognosis, and etiologic diagnosis [37–39]. Clinical management in cases of ovotesticular DSD depends on the patient's age at diagnosis and data of the internal and external genitalia, as in most cases of genital ambiguity. When diagnosed at early ages, the best option is female sex of rearing, trying to maintain the ovarian portion of the gonads when possible, regarding the possibility of spontaneous female puberty and fertility, especially in patients with chromosomal 46,XX constitution [38]. Cases of 46,XX ovotesticular DSD and 46,XX testicular DSD were seen in the same family, in monozygotic twins, suggesting that these two disorders can have one etiology with a broad phenotypic spectrum [40]. Less frequent etiologies of DGD in this series were 46,XX testicular DSD [41] and testicular regression syndrome [42, 43].

Furthermore, there were 19 that were evaluated by presenting a genital complex malformation (not ambiguous genitalia) especially associated with vertebral, urinary, and intestinal tract alterations [44]. Abnormal development of external genitalia may be an isolated anomaly but can also be part of abnormalities in the development of the lower abdominal wall or perineum. As described by Cox et al. [10] and Hutson et al. [15], the investigation of DSD defects in these patients is essential and possibly more often necessary than described in the literature.

Sex assignments before referral to our service and before etiological investigation, in addition to older age at the first visit to our hospital (average of 31.7 months), show that pediatricians need to understand better the concept of DSD and its etiologies as well as clinical and psychosocial implications [45]. The average age at first visit was significantly lower in the 46,XX ovarian DSD group, which may be explained by the large number of cases of 21*α*-hydroxylase deficiency in the salt-wasting form, for whom the risk of death is high if it is not early diagnosed. Only 27 cases (6.6%) have sex reassignment, 15 from male to female (mainly cases of congenital adrenal hyperplasia, mixed gonadal dysgenesis, and 46,XX ovotesticular DSD) and 12 from female to male (mainly 5-alpha-reductase type 2 deficiency). Sex reassignment occurred in the first year of life in all cases, except for patients with 5*α*-reductase type 2 deficiency. About half of the cases of sex reassignment were due to the possibility of normal female puberty and fertility in patients with congenital adrenal hyperplasia (seven cases) and to personal request of adolescents with 5*α*-reductase type 2 deficiency (seven cases), as recommended by Consensus of DSD [1, 2]. In patients with genital ambiguity due to mixed gonadal dysgenesis or ovotesticular DSD, sex definition is a challenge involving prognosis about adult gender identity, anticipated quality of sexual function, surgical options and risks, fertility potential, evidence of fetal CNS exposure to androgens, gonadal malignancy risk, and psychosocial factors (familial, social, and cultural) [1, 2]. In this present study, five patients with mixed gonadal dysgenesis and four patients with ovotesticular DSD (three with 46,XX karyotype e and one with 46,XY karyotype) had sex reassignment, based on surgical options (severity of genital ambiguity), presence of normal uterus, and decision of the family.

Overall, the predominant sex assigned was male (238 cases), following the increased frequency of karyotype 46,XY and the high number of cases of 46,XY testicular DSD.

## 5. Conclusions

With the experience of treating and following 408 DSD patients with genital ambiguity over 23 years in an interdisciplinary care center, we may conclude that individuals with genital ambiguity represent an urgent problem that must be solved quickly and accurately, as early as possible. The management of these patients requires empathy and should be performed by a skilled multidisciplinary team, in order to reach a correct diagnosis without confusion about the child's sexual identification. Making a diagnosis properly before the sex assignment in most cases gives the patient and the family a better understanding of the condition and the proper definition of gender of rearing and allows more satisfaction with treatment. This study with a large sample shows that it is essential for the general pediatricians, who are the first professionals to evaluate the child, to have at least basic knowledge of DSD. Inadequate sex assignments and ineffective treatments may occur if these rare conditions are not properly evaluated.

It can also be concluded that the 46,XY karyotype was more frequent among cases of DSD whereas congenital adrenal hyperplasia was the most common etiology of monogenic inheritance. Cases of 46,XY DSD and DGD require much attention and accurate laboratorial tests for the correct etiological diagnosis. Other malformations were commonly associated in all DSD groups. Several cases without etiologic diagnosis or with syndromic features need advanced technique like next generation sequencing, with either panels of candidate genes or whole exome, making it possible to clarify the etiologic diagnosis and to discover new candidate genes, mainly in 46,XY testicular DSD. Low birth weight was associated with the 46,XY testicular DSD. The average age at the first visit was 31.7 months and was lower in the 46,XX DSD group. The final sex of rearing was male in 238 cases and female in 170, and only 6.6% had sex reassignment.

Finally, we want to emphasize the importance of knowing the prevalence of each etiologic diagnosis of DSD with ambiguous genitalia and the use of both karyotype and gonadal tissue in the classification of DSD groups.

## Supplementary Material

The supplementary materials show the diagnosis criteria of each etiology of DSD included in this study.

## Figures and Tables

**Table 1:** Frequencies of etiological diagnosis in 408 consecutive cases of DSD with ambiguous genitalia followed at GIEDDS, UNICAMP, between January 1989 and December 2011.

Syndromic diagnosis	Etiologic diagnosis	*N*	*F*1	*F*2
46,XX ovarian DSD	Congenital adrenal hyperplasia	69	16.9	65.7
	Isolated clitoromegaly	19	4.7	18.1
	Syndromic	10	2.5	9.5
	Teratogenic^1^	2	0.5	1.9
	Idiopathic	5	1.2	4.8

Total		105	25.8	100.0

46,XY testicular DSD	Hypogonadotropic hypogonadism	12	2.9	6.3
	Defect in the LH/hCG receptor	2	0.5	1.1
	Synthesis of testosterone defect	4	1.0	2.1
	Androgen insensitivity			
	Total	15	3.7	7.9
	Partial	10	2.5	5.3
	5*α*-Reductase type 2 deficiency	20	4.9	10.6
	Muller duct persistence	4	1.0	2.1
	Teratogenic^1^	5	1.2	2.6
	Syndromic	40	9.8	21.2
	Idiopathic	77	18.8	40.8

Total		189	46.3	100.0

Disorders of gonadal development	Ovotesticular DSD	22	5.4	23.1
	Mixed gonadal dysgenesis	25	6.1	26.3
	Partial gonadal dysgenesis			
	*SRY *mutation	2	0.5	2.1
	*WT1* mutation	5	1.2	5.3
	*NR5A1 *mutation	9	2.3	9.5
	No defined cause	22	5.4	23.1
	46,XX testicular DSD	3	0.7	3.2
	Testicular regression syndrome	7	1.7	7.4

Total		95	23.3	100.0

Others	Epispadias	4	1.0	21.0
	Penis malformation	5	1.2	26.3
	Clitoris malformation	6	1.5	31.7
	Multiple malformations	4	1.0	21.0

Total		19	4.7	100.0

*F*1 = relative frequency (%) in relation to the total number of cases of genital ambiguity (408); *F*2 = relative frequency (%) in the number of cases of the diagnostic group (105 46,XX ovarian DSD; 189 46,XY testicular DSD; 95 DDG and 19 others); ^1^  = use of drugs by the mother during pregnancy.

**Table 2:** Frequencies of initial and final sex distributed according to aetiologic diagnosis of 408 consecutive cases of DSD with ambiguous genitalia followed at GIEDDS, UNICAMP, between January 1989 and December 2011.

	Etiologic diagnosis	Age ≤ 6 months	Initial sex			Final sex	
			M	F	I	M	F
46,XX ovarian DSD	Congenital adrenal hyperplasia	50	7	36	26	0	69
	Isolated clitoromegaly	14	0	10	9	0	19
	Teratogenic	1	0	2	0	0	2
	Syndromic	6	0	7	3	0	10
	Idiopathic	3	0	3	2	0	5

46,XY testicular DSD	Hypogonadotropic hypogonadism	4	12	0	0	12	0
	Defect in the LH/hCG receptor	0	0	2	0	0	2
	Defective synthesis of testosterone	1	1	3	0	1	3
	Androgen insensitivity						
	Total	2	0	14	1	0	15
	Partial	4	9	0	1	9	1
	5*α*-Reductase type 2 deficiency	5	2	12	6	13	7
	Muller duct persistence	1	4	0	0	4	0
	Teratogenic	3	4	0	1	5	0
	Syndromic	21	29	1	10	39	1
	Idiopathic	33	57	1	19	76	1

Disorders of gonadal development	Ovotesticular DSD	8	13	4	5	10	12
	Mixed gonadal dysgenesis	13	17	1	7	17	8
	Partial gonadal dysgenesis						
	*SRY* mutation	1	0	0	2	2	0
	*WT1* mutation	2	1	2	2	3	2
	*NR5A1* mutation	4	4	2	3	8	1
	Undefined cause	8	11	3	8	18	4
	46,XX testicular DSD	1	2	0	1	3	0
	Testicular regression syndrome	0	6	1	0	6	1

Others	Epispadias	2	4	0	0	4	0
	Penis malformation	2	3	0	2	5	0
	Clitoris malformation	2	0	5	1	0	6
	Multiple malformations	2	2	0	2	3	1

Total		193	189	108	111	238	170

**Table 3:** Variation of the age at the first visit for 408 consecutive cases of DSD with ambiguous genitalia followed at GIEDDS, UNICAMP, between January 1989 and December 2011.

Diagnostic	*n*	Age (months)				
		Mean	Median	SD	Minimum	Maximum
46,XX ovarian DSD	105	15.9	1.7	33.7	0.0	173.0
46,XY testicular DSD	189	35.4	13.7	60.1	0.1	301.0
Disorders of gonadal differentiation	95	45.3	9.0	69.3	0.2	324.0
Others	19	14.8	8.1	22.4	0.3	96.2

Total	408	31.7	7.0	56.7	0.0	324.0

## References

[1] Lee P. A., Houk C. P., Ahmed S. F. (2006). Consensus statement on management of intersex disorders. International Consensus Conference on Intersex. *Pediatrics*.

[2] Lee P. A., Nordenström A., Houk C. P. (2016). Global disorders of sex development update since 2006: perceptions, approach and care. *Hormone Research in Paediatrics*.

[3] Palmer B. W., Wisniewski A. B., Schaeffer T. L. (2012). A model of delivering multi-disciplinary care to people with 46 XY DSD. *Journal of Pediatric Urology*.

[4] Fausto-Sterling A. (2000). *Sexing the Body: Gender Politics and the Construction of Sexuality*.

[5] Sax L. (2002). How common is intersex? A response to Anne Fausto-Sterling. *Journal of Sex Research*.

[6] Pasterski V., Prentice P., Hughes I. A. (2010). Impact of the consensus statement and the new DSD classification system. *Best Practice and Research: Clinical Endocrinology and Metabolism*.

[7] Aaronson I. A., Aaronson A. J. (2010). How should we classify intersex disorders?. *Journal of Pediatric Urology*.

[8] Houk C. P., Lee P. A. (2012). Update on disorders of sex development. *Current Opinion in Endocrinology, Diabetes and Obesity*.

[9] Romao R. L. P., Salle J. L. P., Wherrett D. K. (2012). Update on the management of disorders of sex development. *Pediatric Clinics of North America*.

[10] Cox K., Bryce J., Jiang J. (2014). Novel associations in disorders of sex development: findings from the I-DSD registry. *Journal of Clinical Endocrinology and Metabolism*.

[11] Rey R. A., Grinspon R. P. (2011). Normal male sexual differentiation and aetiology of disorders of sex development. *Best Practice and Research: Clinical Endocrinology and Metabolism*.

[12] Al-Mutair A., Iqbal M. A., Sakati N., Ashwal A. (2004). Cytogenetics and etiology of ambiguous genitalia in 120 pediatric patients. *Annals of Saudi Medicine*.

[13] Gidlöf S., Wedell A., Guthenberg C., Von Döbeln U., Nordenström A. (2014). Nationwide neonatal screening for congenital adrenal hyperplasia in sweden a 26-year longitudinal prospective population-based study. *JAMA Pediatrics*.

[14] Guaragna-Filho G., Castro C. C. T. D. S., De Carvalho R. R. (2012). 46,XX DSD and Antley-Bixler syndrome due to novel mutations in the cytochrome P450 oxidoreductase gene. *Arquivos Brasileiros de Endocrinologia e Metabologia*.

[15] Hutson J. M., Grover S. R., O'Connell M., Pennell S. D. (2014). Malformation syndromes associated with disorders of sex development. *Nature Reviews Endocrinology*.

[16] Veiga-Junior N. N., Medaets P. A. R., Petroli R. J. (2012). Clinical and laboratorial features that may differentiate 46,XY DSD due to partial androgen insensitivity and 5*α*-reductase type 2 deficiency. *International Journal of Endocrinology*.

[17] Hackel C., Oliveira L. E. C., Ferraz L. F. C. (2005). New mutations, hotspots, and founder effects in Brazilian patients with steroid 5*α*-reductase deficiency type 2. *Journal of Molecular Medicine*.

[18] Hackel C., Oliveira L. E., Toralles M. B. (2005). 5*α*-reductase type 2 deficiency: experiences from Campinas (SP) and Salvador (BA). *Arquivos Brasileiros de Endocrinologia e Metabologia*.

[19] Stuchi-Perez E. G., Hackel C., Oliveira L. E. C. (2005). Diagnosis of 5*α*-reductase type 2 deficiency: contribution of anti-Müllerian hormone evaluation. *Journal of Pediatric Endocrinology and Metabolism*.

[20] Choi J.-H., Kim G.-H., Seo E.-J., Kim K.-S., Kim S. H., Yoo H.-W. (2008). Molecular analysis of the AR and SRD5A2 genes in patients with 46,XY disorders of sex development. *Journal of Pediatric Endocrinology and Metabolism*.

[21] Cohen-Kettenis P. T. (2005). Gender change in 46,XY persons with 5*α*-reductase-2 deficiency and 17*β*-hydroxysteroid dehydrogenase-3 deficiency. *Archives of Sexual Behavior*.

[22] Grumbach M. M. (2005). Commentary: a window of opportunity: the diagnosis of gonadotropin deficiency in the male infant. *Journal of Clinical Endocrinology and Metabolism*.

[23] De Andrade Machado Neto F., Moreno Morcillo A., Trevas Maciel-Guerra A., Guerra-Junior G. (2005). Idiopathic male pseudohermaphroditism is associated with prenatal growth retardation. *European Journal of Pediatrics*.

[24] Nef S., Verma-Kurvari S., Merenmies J. (2003). Testis determination requires insulin receptor family function in mice. *Nature*.

[25] Ostrer H. (2014). Disorders of sex development (DSDs): an update. *The Journal of Clinical Endocrinology & Metabolism*.

[26] Scolfaro M. R., Cardinalli I. A., Stuchi-Perez E. G. (2001). Morphometry and histology of gonads from 13 children with dysgenetic male pseudohermaphroditism. *Archives of Pathology and Laboratory Medicine*.

[27] dos Santos A. P., Ribeiro Andrade J. G., Piveta C. S. C. (2013). Screening of Y chromosome microdeletions in 46,XY partial gonadal dysgenesis and in patients with a 45,X/46,XY karyotype or its variants. *BMC Medical Genetics*.

[28] Assumpção J. G., Benedetti C. E., Maciel-Guerra A. T. (2002). Novel mutations affecting SRY DNA-binding activity: the HMG box N65H associated with 46,XY pure gonadal dysgenesis and the familial non-HMG box R30I associated with variable phenotypes. *Journal of Molecular Medicine*.

[29] Assumpção J. G., Ferraz L. F. C., Benedetti C. E. (2005). A naturally occurring deletion in the SRY promoter region affecting the Sp1 binding site is associated with sex reversal. *Journal of Endocrinological Investigation*.

[30] De Andrade J. G. R., Guaragna M. S., Soardi F. C., Guerra G., De Mello M. P., Andréa T. M.-G. (2008). Clinical and genetic findings of five patients with WT1-related disorders. *Arquivos Brasileiros de Endocrinologia e Metabologia*.

[31] Guaragna M. S., Lutaif A. C. G. D. B., Bittencourt V. B. (2012). Frasier syndrome: four new cases with unusual presentations. *Arquivos Brasileiros de Endocrinologia e Metabologia*.

[32] Fabbri H. C., de Andrade J. G. R., Soardi F. C. (2014). The novel p.Cys65Tyr mutation in NR5A1 gene in three 46,XY siblings with normal testosterone levels and their mother with primary ovarian insufficiency. *BMC Medical Genetics*.

[33] Soardi F. C., Coeli F. B., Maciel-Guerra A. T., Guerra-Júnior G., Palandi de Mello M. (2010). Complete XY gonadal dysgenesis due to p.D293N homozygous mutation in the NR5A1 gene: a case study. *Journal of Applied Genetics*.

[34] Loureņo D., Brauner R., Lin L. (2009). Mutations in NR5A1 associated with ovarian insufficiency. *The New England Journal of Medicine*.

[35] de Andrade J. G. R., Guerra-Júnior G., Maciel-Guerra A. T. (2010). 46,XY and 45,X/46,XY testicular dysgenesis: similar gonadal and genital phenotype, different prognosis. *Arquivos Brasileiros de Endocrinologia e Metabologia*.

[36] Johansen M. L., Hagen C. P., Rajpert-De Meyts E. (2012). 45,X/46,XY mosaicism: phenotypic characteristics, growth, and reproductive function-a retrospective longitudinal study. *Journal of Clinical Endocrinology and Metabolism*.

[37] Guerra-Junior G., De Mello M. P., Assumpção J. G. (1998). True hermaphrodites in the southeastern region of Brazil: a different cytogenetic and gonadal profile. *Journal of Pediatric Endocrinology and Metabolis*.

[38] Sircili M. H., Denes F. T., Costa E. M. (2014). Long-term followup of a large cohort of patients with ovotesticular disorder of sex development. *The Journal of Urology*.

[39] Matsui F., Shimada K., Matsumoto F. (2011). Long-term outcome of ovotesticular disorder of sex development: a single center experience. *International Journal of Urology*.

[40] Maciel-Guerra A. T., De Mello M. P., Coeli F. B. (2008). XX Maleness and XX true hermaphroditism in SRY-negative monozygotic twins: additional evidence for a common origin. *The Journal of Clinical Endocrinology & Metabolism*.

[41] Damiani D., Guedes D. R., Damiani D. (2005). XX male: 3 case reports during childhood. *Arquivos Brasileiros de Endocrinologia e Metabologia*.

[42] Maciel-Guerra A. T., Farah S. B., Garmes H. M. (1991). True agonadism: report of a case analyzed with Y-specific DNA probes. *American Journal of Medical Genetics*.

[43] Pirgon Ö., Dündar B. N. (2012). Vanishing testes: a literature review. *Journal of Clinical Research in Pediatric Endocrinology*.

[44] Guerra-Junior G., Aun A. M. E., Miranda M. L. (2008). Congenital perineal lipoma presenting as ambiguous genitalia. *European Journal of Pediatric Surgery*.

[45] Guerra-Júnior G., Maciel-Guerra A. T. (2007). The role of the pediatrician in the management of children with genital ambiguities. *Jornal de Pediatria*.

