# Supplementary material for: 408 Cases of Genital Ambiguity Followed by Single Multidisciplinary Team during 23 Years: Etiologic Diagnosis and Sex of Rearing
Source: Int J Endocrinol. 2016 Nov 28;2016:4963574. doi: 10.1155/2016/4963574 (PMC5149677; doi:10.1155/2016/4963574)
Supplement: Supplementary file 1 — The supplementary materials show the diagnosis criteria of each etiology of DSD included in this study. [file 4963574.f1.doc]

**Supplement**

The DGD included cases of ovotesticular DSD, mixed gonadal dysgenesis, XY partial gonadal dysgenesis, 46,XX testicular DSD and 46,XY testicular regression. 46,XX ovarian DSD included cases of congenital adrenal hyperplasia, isolated clitoromegaly, drugs used by the mother during pregnancy (teratogenic), malformations (syndromic) and unclear (idiopathic). The 46,XY testicular DSD included cases of hypogonadotropic hypogonadism, isolated or with hypopituitarism, a defect in the LH/hCG receptor, defect in testosterone synthesis with or without congenital adrenal hyperplasia, defect in the androgen receptor in full and partial forms, 5α-reductase type 2 deficiency, drugs used by the mother during pregnancy (teratogenic), malformations (syndromic) and unclear (idiopathic). The cases of complex malformations of the external genitalia (other) included epispadias, malformation of the penis or the clitoris and other complex malformations including abnormalities of the external genitalia.

For the definition of each aetiology the following criteria were established: (I) independent of karyotype: ovotesticular DSD: presence of testicular tissue (seminiferous tubules) and ovarian (follicles) in the same individual; (II) mixed gonadal dysgenesis: karyotype with 45,X lineage and lineage containing Y with absence of ovarian tissue in the gonads and presence of bilateral gonadal dysgenesis (different associations between testicular dysgenesis; undifferentiated gonadal tissue and streak gonad); (III) 46,XY karyotype: (III.1) partial gonadal dysgenesis: presence of dysgenetic gonads and Müllerian ducts (in these cases, *SRY*, *WT1*, *NR5A1* and *SOX9* genes were evaluated); (III.2) testicular regression: macro and microscopic absence of testicular tissue without Müllerian ducts; (III.3) hypogonadotropic hypogonadism (isolated or with hypopituitarism): presence of a micropenis with or without cryptorchidism without hypospadias with low levels of LH; (III.4) defect in LH receptor/hCG: defect in testosterone production without an increase of the adrenal precursors, absence of Leydig cells in the gonadal biopsy and *LHCGR* mutation; (III.5) defect of testosterone synthesis with or without congenital adrenal hyperplasia: defect in testosterone production to increase at least one of the adrenal precursors (DHEA, progesterone or androstenedione) and mutation in the specific gene (*HSD3B2*, *CYP17A1*, *HSD17B3* genes); (III.6) defect in the androgen receptor (partial or total): normal production of testosterone, with or without increasing LH and mutation in *AR* gene; (III.7) 5α-reductase type 2 deficiency: normal testosterone production without increasing dihydrotestosterone (DHT) with mutation in *SRD5A2* gene; (III.8) persistence of Müllerian ducts: the presence of uterine and/or Fallopian tubes and no other changes in the internal and external male genitalia and mutation in *AMH* or *AMHR* gene; (III.9) teratogenic: declared use of drugs by the mother during pregnancy with normal testosterone production; (III.10) syndromic: presence of genital ambiguity associated with malformations without aberration of sex chromosomes; (III.11) idiopathic or unclear: genital ambiguity with normal testosterone production without associated malformations or use of drugs during pregnancy, normal molecular study of *AR*, *SRD5A2* and *NR5A1* genes; (III.12) malformation of the penis: malformation (not ambiguous genitalia) of the penis; (III.13) epispadias: urethral opening on the dorsal surface of the penis with or without associated urinary malformation; (III.14) multiple and complex malformation: genital malformation (and not ambiguous genitalia) associated with other urinary, intestinal and/or vertebral malformations; (IV) karyotype 46,XX: (IV.1) 46,XX testicular DSD: presence of bilateral testicular tissue; (IV.2) congenital adrenal hyperplasia: increasing of one of the precursors of adrenal biosynthesis (17-OH progesterone, 11-deoxycortisol, progesterone, DHEA) with decreasing cortisol and increasing ACTH and mutation in the specific gene (*CYP21A2*, *POR*); (IV.3) isolated clitoromegaly: exclusive increasing the clitoris and absence of any hormonal alteration; (IV.4) teratogenic: declared use of drugs by the mother during pregnancy with normal hormone production; (IV.5) syndromic: presence of genital ambiguity associated with malformation features without aberration of sex chromosomes with normal hormone production; (IV.6) idiopathic or unclear: genital ambiguity frame with normal hormone production without associated malformations or use of drugs during pregnancy; (IV.7) malformation of the clitoris: malformation (not ambiguous genitalia) of the clitoris; (IV.8) multiple and complex malformation: genital malformation (not ambiguous genitalia) associated with other urinary, intestinal and/or vertebral malformations.
